# MiR-206 may regulate mitochondrial ROS contribute to the progression of Myocardial infarction via TREM1

**DOI:** 10.1186/s12872-023-03481-8

**Published:** 2023-09-20

**Authors:** Hao Lin, Jiapeng Chu, Deqiang Yuan, Kangwei Wang, Fei Chen, Xuebo Liu

**Affiliations:** Department of Cardiology, Tongji Hospital, School of Medicine, Tongji University, No.389, Xincun Road, Shanghai, 200092 Putuo District China

**Keywords:** Myocardial infarction, Bioinformatics, Hub genes, MiRNA, Mitochondrial ROS

## Abstract

Myocardial infarction (MI) is a leading cause of mortality. To better understand its molecular and cellular mechanisms, we used bioinformatic tools and molecular experiments to explore the pathogenesis and prognostic markers. Differential gene expression analysis was conducted using GSE60993 and GSE66360 datasets. Hub genes were identified through pathway enrichment analysis and PPI network construction, and four hub genes (AQP9, MMP9, FPR1, and TREM1) were evaluated for their predictive performance using AUC and qRT-PCR. miR-206 was identified as a potential regulator of TREM1. Finally, miR-206 was found to induce EC senescence and ER stress through upregulating mitochondrial ROS levels via TREM1. These findings may contribute to understanding the pathogenesis of MI and identifying potential prognostic markers.

**Supplementary Information:**

The online version contains supplementary material available at 10.1186/s12872-023-03481-8.

## Introduction

According to the review from China, cardiovascular disease (CVD) is the main cause of death in China, it is significant to explore the mechanisms and features of CVD to cope with the challenges of CVD epidemics [1]. MI is a common, leading cause of morbidity and mortality in CVD [2]. Acute coronary syndrome (ACS) including ST elevation myocardial infarction (STEMI), unstable angina (UA) and non-ST elevation myocardial infarction (NSTEMI) [3]. The pathological mechanism of STEMI is complex and multifactorial, the primary pathogenesis is plaque rupture and subsequently thrombus-induced myocardial necrosis [4].

Ischemic time duration and the early execution of the best reperfusion strategy for patients with a STEMI are crucial determinants of infarct size. As a result, prompt recognition and diagnosis are critical to slowing the progression of STEMI and improving patient survival and prognosis in the hospital [5, 6]. A valid early biomarker might contribute to the diagnosis of STEMI, especially in the early pathological stages. It is generally known that discovering the biomarker helps us better understand the many cellular and molecular pathways of illness development. Currently, there are only a few validated biomarkers, which consist of troponin T (TnT) and CK-MB, which have been applied clinically and are both markers of myocardial cell lysis. But on the other hand, mitochondria, as the major modifier of apoptosis, also play an important role in cardiomyocyte necrosis [7]. HtrA2, for example, a potential novel biomarker of mitochondrial associated reperfusion cardiomyocyte apoptosis and increased in patients with STEMI after percutaneous coronary intervention (PCI) [8]. Likewise, inflammation, thrombosis, and atherosclerosis (AS) participate in accelerating STEMI progression, and research has shown that serum sulfatides correlate positively with in-hospital mortality and complications in STEMI patients [9]. A study about (microRNA) miRNA suggests that miR-499-5p can be an independent predictor of STEMI after adjustment for risk factors by measuring plasma miRNA in STEMI patients [10]. It is indicated that miRNAs act as regulators of gene expression and can influence many essential cellular processes, which play a critical role in the pathogenesis of multiple chronic diseases, including STEMI and AS. Furthermore, plasma HSP70 has been indicated to predict the early prognosis of STEMI [11]. Endothelial cells (ECs) also take part in regulating the procession of AS and STEMI. Endothelium can play a variety of roles in vascular permeability, leukocyte infiltration, vasomotor function, and angiogenesis [12]. The rise of ECs injury-associated biomarkers in plasma correlates with the poor prognosis of vascular disease [12]. Yet, only a few longitudinal investigations have looked at the relationship between EC interactions and extracellular and intracellular processes.

MiRNAs are a class of endogenous small non-coding RNAs known for their role in negatively regulating the expression level of target genes [13]. The miRNAs’ tissue-specific expression pattern is necessary for the development of various diseases, including autoimmunity, cancer, and CVD, among others [14, 15]. Circulating miRNAs have been found to serve as competent biomarkers for various diseases. Due to the above biological features of miRNAs, the expression profile of circulating miRNAs in plasma may reflect the mechanism of progress in CVD, especially in MI. In previous studies, miR-208a, miR-499, miR-133, and miR-127 in plasma were identified as biomarkers for acute myocardial infarction (AMI) patients [16]. And further studies have found miR-208 regulates cardiomyocyte apoptosis through the Bcl-2 [17] and Bax [18] signaling pathways. However, it is not clear how miRNA regulates the function of arterial ECs in the occurrence of myocardial infarction (MI) and needs further research.

In this study, therefore, we analyzed the STEMI data in the GEO database. And we use two GEO data sets involving 57 samples investigated in two conditions. We first identified the key DEGs for each dataset. Subsequently, the DEGs of each dataset were subjected to the Kyoto Encyclopedia of Genes and Genomes (KEGG) and GO enrichment analysis, and the crucial hub genes were found based on the PPI network. In addition, we reverse-predicted the upstream miRNAs of hub genes through web-based miRNA databases. Then, RNA was isolated from the peripheral blood of STEMI patients to validate the expression conditions of hub genes and hub miRNAs by qRT-PCR. Finally, we explore the possible underlying mechanisms of hub miRNA regulation on endothelial dysfunction. Our findings shed light on the pathophysiology (especially endothelial dysfunction) of MI and its biomarkers.

## Materials & methods

### Screen and download the datasets

**Fig. 1:**
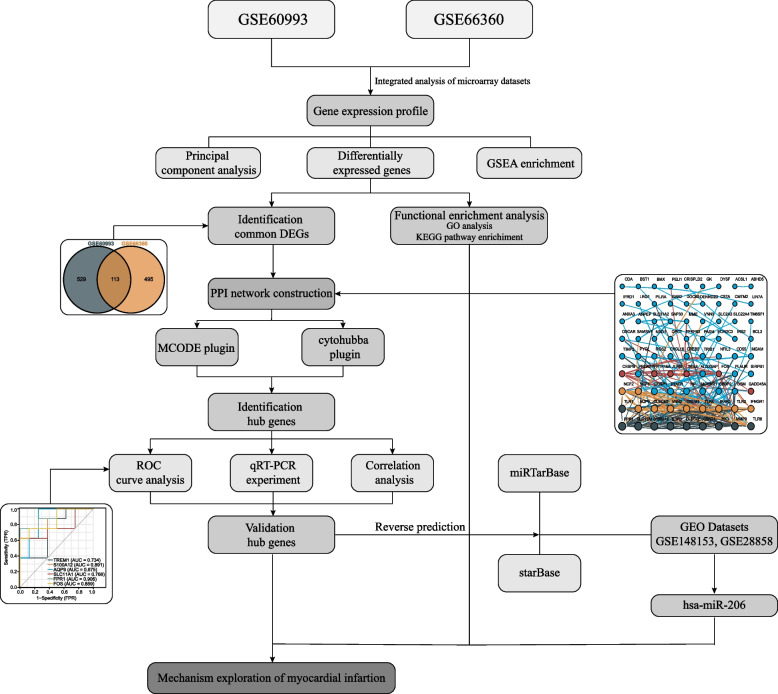
The workflow of the integrative transcriptomics analyses and experimental validation

We searched using terms consisting of "STEMI" or "ACS" and selected the datasets that did match the requirements for us, then downloaded them from the NCBI GEO database (https://www.ncbi.nlm.nih.gov/geo/). We obtained six datasets: GSE66360, GSE60993, GSE103182, GSE123342, GSE141512, and GSE166780, for the analysis and verification of hub genes related to MI. We paired GSE60993 and GSE66360 and performed DEG analysis on them as the discovery cohort. The remaining four datasets are used as validation datasets to evaluate the performance of the discovery cohort. GSE 66360, we collected samples from patients experiencing acute MI (*n* = 49) and from healthy cohorts (*n* = 50), and we picked 43 samples among them into the discovery cohort for follow-up analysis. And measured gene expression using the GPL570 platform (Affymetrix Human Genome U133 Plus 2.0 Array). Then we selected 7 STEMI patients’ peripheral blood samples and 7 control samples from GSE60993, which were measured by GPL6884 (Illumina HumanWG-6 v3.0 expression beadchip). See Table 1 for additional details. Evaluation of the prognostic value of hub miRNAs was carried out by ROC analysis in GSE148153 and GSE28858. The overall screening methods and analysis process of this study are presented in Fig. 1.

**Table 1:** Microarray information

GEO accession	GSE60993	GSE66360
Platform	GPL6884 Illumina HumanWG-6 v3.0	GPL570 Affymetrix HU133 Plus 2.0
Country	South Korea	American
Research object	Human	Human
Probe number	48,803	54,675
AMI	7	49
Control sample	7	50
Public time	Sep 02, 2014	Feb 27, 2015

### GEO data processing and identification of DEGs and consensus genes between the discovery cohort

GSE66360 and GSE60993 datasets were downloaded using the GEO query package [19]. The data normalization analysis and DEGs identification were performed using the R package limma (v3.46.0) [20].^.^ Then, we converted the probe ID to gene symbol. When multiple probe sets were mapped to the same gene symbol, the expression values of these probes were averaged. Based on the comparison conditions between the experimental and control groups, DEGs cut-off criteria were obtained using an adjusted *p*-value (adj.p) ≤ 0.05 and the absolute log2(foldchange) ≥ 0.7. The consensus genes between GSE66360 and GSE60993 datasets were displayed in the Venn diagram.

### Functional enrichment analysis

GO is a systematically annotated-based method and process for genes and their product attributes. It mainly included biological processes, molecular function, and cellular compartments [21]. The purpose of performing GO enrichment analysis is to understand the main activities of gene products at the molecular level and the function of genes in cells. KEGG pathway enrichment analysis is usually used to understand the molecular interaction network in cells and the unique changes of specific organisms [22]. And we mapped all DEGs and found enriched terms in the KEGG database. GSEA can be used as a tool to identify classes of overexpressed genes and the possible disease phenotypes related to these genes [23]. To analyze the GO terms, KEGG pathways and GSEA of DEGs of GSE60993 and GSE66360 respectively was performed using the "clusterProfiler" package [24] and "org.Hs.eg.db" package from R Bioconductor and displayed using “GOplot” package [25]. And in order to visualize the functional enrichment analysis, the “enrichplot” and “gseaplot2” packages were performed [24]. *P* < 0.05 was considered statistically significant.

### PPI network construction and crucial module analysis

The PPI network analysis can help us to understand new insights and complementary information for the prediction of protein function. It may contribute to providing the principles of functional cellular networks [26]. So, the STRING (V11.5, https://string-db.org/) online database was used for the construction and download of the human PPI network of the DEGs [27]. And the confidence score ≥ 0.4 was selected to construct the PPI network, it was considered the medium confidence score. Then, Cytoscape (bioinformatic software, V3.8.2) was used for the visualization of the PPI network, and each node in the network denotes a gene or protein, while the edge represents the interactions of the molecules [28].

Based on the analysis of previous studies genome-wide, the alteration of hub genes is more likely to be lethal than the alteration of non-hub genes. Therefore, it is crucial to find the hub genes. MCODE (Molecular Complex Detection, V1.5.1) is a Cytoscape plug-in that employs topological analysis to identify densely connected areas [29]. The criteria for the MCODE analysis to pick the modules were that the MCODE score > 5, the degree of cutoff = 2, node score cutoff = 0.2, Max depth = 100, and k-score = 2.

### Hub genes analysis and construction of the disease prediction model

The degree of node indicates the number of interactions between one gene and other genes. We consider that the genes with higher degrees of interaction can act as hub genes. Then, we used the cytoHubba plugin in Cytoscape to filter top 10 genes of DEGs based on the Maximal Clique Centrality (MCC) algorithm [30]. Finally, to select 10 hub genes based on comprehensive factors such as MCODE score and degree ranking. The Pearson correlation coefficient was used to perform the correlation of hub gene analysis and was shown using the corrplot package in R in order to better understand the relationship between hub genes and the biological processes they are involved in. Furthermore, to assess the diagnostic function of hub genes in MI, receiver operating characteristic (ROC) curves were performed in validation datasets and visualized using the pROC package in R, and the AUC was calculated.

### Cell culture

The human umbilical vein endothelial cells (HUVECs) line was purchased from the Chinese Academy of Sciences (category number: EAhy926), and was grown in Dulbecco’s Modified Eagle’s Medium (low glucose, DMEM) from Sigma (Sigma-Aldrich, MO, USA, D6046) with 10% fetal bovine serum (FBS, Sigma-Aldrich, MO, USA, F8318) and penicillin–streptomycin (100 U/mL, Gibco) at 37 °C with a 5% CO2 supplied incubator.

### Transfections

Hsa-miR-206 mimics and miRNA mimic negative control were purchased from Genomeditech company (Shanghai, China). 24 h before transfection, cells were seeded at 1 × 10^5^ cells per well of 6-well plates in antibiotic free medium. Lipofectamine 3000 (Invitrogen, CA) and opti-MEM (Gibco, cat.31985062) medium reagents were used for transfection according to the manufacturer's instructions. And the miRNA mimics and negative control were used at a final concentration of 50 nM according to the manufacturer's instructions. For overexpression and knockdown of TREM1, adenovirus (Adv) and lentivirus (LV) were used for cell transfection. Adv-TREM1 and LV-TREM1 (Hanheng Biotechnology, Shanghai, China) were directly added to cultured cells and incubated for 72 h. Adv and LV expressing GFP were used as controls.

### Validation of expression at the mRNA level by qPCR

The validation of hub gene expression was conducted using qPCR. Eight peripheral blood samples from STEMI patients who underwent percutaneous coronary intervention (PCI) and two samples from matched patients with angiogram-negative were collected in 2021 at Tongji Hospital of Shanghai (this study was executed in accordance with the Declaration of Helsinki). The diagnosis of STEMI is based on the presence of typical chest pain, symptoms of myocardial ischemia, elevation of cardiac biomarkers (troponin T/I or creatine kinase-MB), and ST-segment elevation > 0.1 mV in at least two contiguous leads or new left bundle branch block on a 12-lead electrocardiogram [31]. The exclusion criteria were: severe autoimmune disease and infectious disease, tumor or have a left ventricular ejection fraction (LVEF) ≤ 30%. No further exclusion criteria were used for control group. Informed consent was waived due to the anonymized processing of patient data, and all of these STEMI and control patients had received coronary angiography. Approval was obtained from the Institutional Ethics Committee of the Tongji Hospital, Tongji University (2021-KYSB-121). In addition, all peripheral blood mononuclear cell (PBMC) were isolated from whole blood samples with a human PBMC separation kit (Solarbio, Beijing, China, Cat. No. P8610) according to the manufacturer's instructions. All PBMC and HUVEC were performed with TRIzol Reagent (Invitrogen, Life Technologies Corporation, USA) and stored at − 80 °C until total RNA extraction. The total RNAs were reverse transcribed using PrimeScript™ RT Master Mix (RR036A, TaKaRa, Dalian, China). And the cDNA was amplified using Taq Pro Universal SYBR qPCR Master Mix (Vazyme Biotech, Nanjing, China, Q712-02) for q-PCR on an Applied Biosystems QuantStudio 5 system (Thermo Fisher Scientific). Results from qPCR were analyzed using the 2-ΔCT quantitative method and the expression level of β-actin or GAPDH were used to normalize the expression level of target genes. The primer sequences used are listed in Table S1.

### Prediction and validation of hub miRNAs related to the progression of MI

To predict candidate miRNAs that target hub genes by using the miRTarBase (Version9.0, https://mirtarbase.cuhk.edu.cn/~miRTarBase/miRTarBase_2022/php/index.php) [32] and starBase (https://starbase.sysu.edu.cn/) [33] databases. In addition, to further identify the important miRNA in MI, the miRNA-diseases associations information was acquired from the Human MicroRNA Disease Database (HMDD, V3.2) [34]. Subsequently, we screened the potential miRNAs by taking the intersection of the top highly connected miRNAs and the MI-associated miRNAs.

Finally, we carried out ROC curves to assess the prognostic value of candidate miRNAs in GSE148153 and GSE28858 and visualized using the pROC package in R. Also, we verified the expression level of miRNAs in PBMC samples from STEMI patients and controls via qPCR experiments.

### Western blotting

Total protein of HUVEC was extracted with RIPA lysis buffer (Epizyme PC101) followed by centrifuged at 12000 rpm for 10 min at 4℃. Total protein of each sample was separated by SDS-PAGE (Epizyme), then transfer to PVDF membranes (Sigma-Aldrich, MO, USA, ISEQ00010) and blocked in 5% nonfat skim milk (Epizyme, PS112) for 2 h at room temperature. After blocking, the membrane was incubated with the following primary antibodies overnight at 4 ℃: GRP78 (Cell Signaling Technology, #3177S, 1:1000), p21 (Cell Signaling Technology, #2946S, 1:1000), Sirt6 (Cell Signaling Technology, #12486S, 1:1000), p-AMPKα (Cell Signaling Technology, #50081S, 1:1000), TREM1 (Abcam, ab200729, 1:1000), GAPDH (SIMUWU, SD0033, 1:1000), β-Tubulin (Abmart, #M30109, 1:1000). and then coated with corresponding HRP-labeled secondary antibodies (SIMUWU, SD0038 (anti-mouse), SD0039 (anti-rabbit)) at room temperature for 1 h after washing three times using PBS 0.1% Tween-20. The immunoblots reaction was detected using an enhanced chemiluminescence reagent (ECL). GAPDH and β-Tubulin was used as the internal control.

### Mitochondrial ROS measurement

Mitochondrial ROS in HUVECs was measured by MitoSOX Red Mitochondrial Superoxide Indicator (Yeasen, #40778ES50, China) according to manufacturer’s instruction. For nuclei staining, Hoechst 33,342 (Yeasen, #40732ES03, China) was used at 5 μg/ml. And then images of fluorescent were observed and collected by Confocal laser-scanning microscopy (Nikon).

### Statistical analysis

All statistical analyses were conducted using the GraphPad Prism 9.0 software. The results are presented as the means ± standard deviation (SDs). In case of multiple comparisons, the data was determined using a one-way ANOVA test. Each experiment was repeated at least twice and *p* values < 0.05 were considered statistically significant.

## Results

### Identification of DEGs and common genes in patients with STEMI

**Fig. 2:**
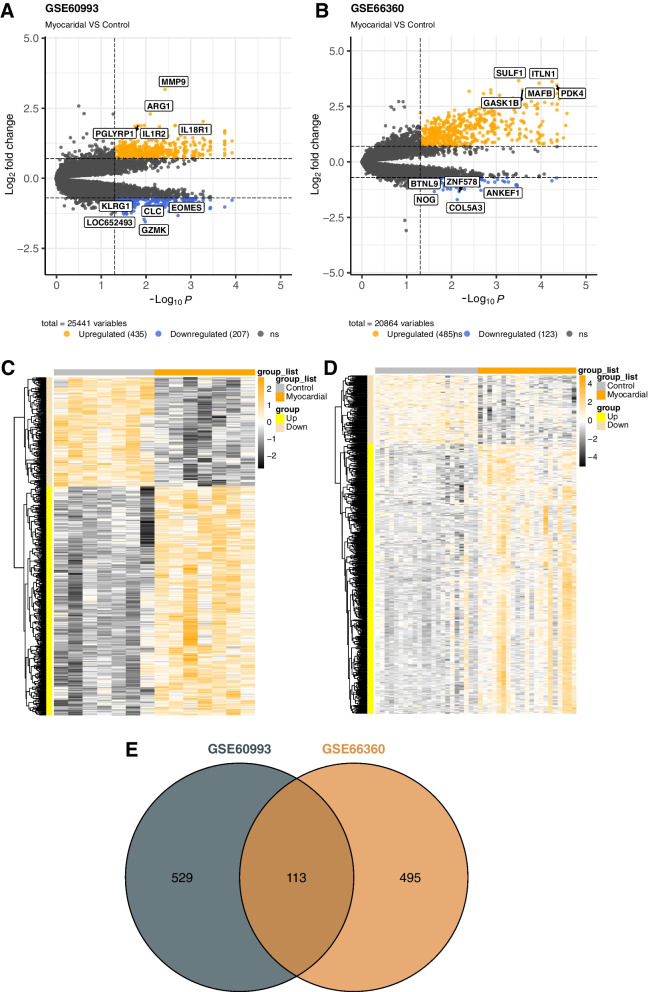
Identification of DEGs. **A**-**B** The volcano plot shows distribution of the DEGs of GSE60993 and GSE66360. And the top 10 differential genes labeled in box. (Orange represents upregulated genes and royalblue represents downregulated genes). **C**-**D** Heatmaps of the DEGs was constructed for GSE60993 and GSE66360 respectively. (Yellow represents upregulated genes and wheat represents downregulated genes; Orange represents experimental groups and grey represents control groups). **E** Venn to identify 113 overlapped DEGs from GSE60993 and GSE66360 datasets. DEGs: Differential expression genes

Principal component analysis (PCA) was used for preliminary data studies and quality control. The samples of two datasets were performed with PCA analysis, and the package "ggfortify" [35] was used for it by R software. Results from the PCA plot show that samples of the two groups in two GEO datasets can be differentiated (Supplementary file, Supplementary Fig. 1). A total of 642 DEGs (435 up, 207 down) in GSE60993 and 608 DEGs (485 up, 123 down) in GSE66360 were identified using the R package limma with the cutoff criterion of adj.*p* value ≤ 0.05 and |log FC|≥ 0.7 (Fig. 2A-D). Moreover, within the identified DEGs, we marked the top 10 of the ranked genes in gene expression from two datasets separately. As shown in Fig. 2, SULF1, ITLN1, MAFB, PDK4, GASK1B, COL5A3, ZNF578, NOG, ANKEF1 and BTNL9 were the top 10 (Upregulated and downregulated) genes in GSE66360 and MMP9, ARG1, IL18R1, IL1R2 and PGLYRP1 in GSE60993. Finally, we extracted 113 overlapped DEGs from GSE60993 and GSE66360 datasets using Venn diagram (Fig. 2E).

### Functional enrichment and pathways mediated by DEGs

**Fig. 3:**
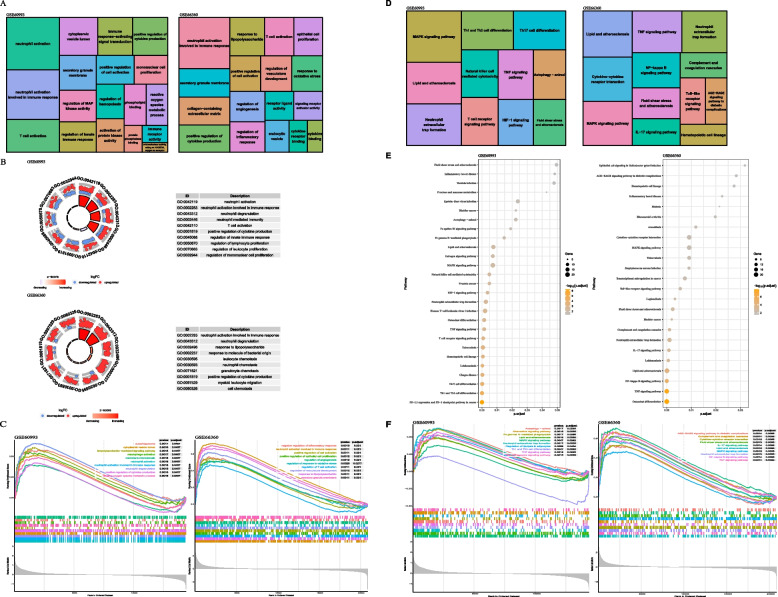
GO functional analysis and KEGG pathway enrichment of DEGs. **A** Treemap plot shows the distribution of GO enrichment results of GSE60993 and GSE66360, the larger of the box area, the larger the counts of enriched gene in the GO terms. **B** The GOcircle plot indicates the gene expression distribution in each GO term and their Z‐score value of GSE60993 and GSE66360. **C** The findings revealed by the GSEA of GSE60993 and GSE66360. **D** Treemap plot shows the distribution of KEGG pathway terms of GSE60993 and GSE66360, the larger of the box area, the larger the counts of enriched gene in the KEGG pathways. **E** KEGG pathway enrichment results of two datasets. **F** The most significant KEGG signaling pathways of GSE60993 and GSE66360 matched in GSEA

Functional enrichment analysis was performed to investigate how the DEGs play key functions and involve disease development. To sum up, it suggested the following. Enrichment analysis revealed these GO terms "neutrophil activation", "neutrophil activation involved in immune response", "positive regulation of cytokine production", "regulation of innate immune response", "reactive oxygen species metabolic process", "regulation of MAP kinase activity" and so forth were significantly enriched in myocardial samples of GSE60993. And "response to lipopolysaccharide", "T cell activation", "epithelial cell proliferation", "positive regulation of cell activation", "regulation of vasculature development", "response to oxidative stress", "regulation of inflammatory response" and so forth were found in GSE66360. Furthermore, "neutrophil activation involved in immune response", "T cell activation", "positive regulation of cell activation", "secretory granule membrane" and so forth inflammation related GO terms both enriched in myocardial samples of two datasets. And, these findings were also supported by evidence from GSEA (Fig. 3A-C).

Then, the DEGs of GSE60993 related to these pathways were used to explore the myocardial signaling network based on the following KEGG pathways: MAPK signaling pathway (ID: hsa04010), Lipid and atherosclerosis (ID: hsa05417), Th1 and Th2 cell differentiation (ID: hsa04658), Neutrophil extracellular trap formation (ID: hsa04613), Natural killer cell mediated cytotoxicity(ID: hsa04650), HIF-1 signaling pathway (ID: hsa04066), Fluid shear stress and atherosclerosis(ID: hsa05418) and other unlisted pathways. And enriched KEGG pathways for DEGs of GSE66360 consisted of Lipid and atherosclerosis (ID: hsa05417), TNF signaling pathway (ID: hsa04668), NF-kappa B pathway (ID: hsa04064), Toll-like receptor signaling pathway (ID: hsa04620), AGE-RAGE signaling pathway in diabetic complications (ID: hsa04933) and other pathways. Among these pathways, there several classical pathways related to inflammation and CVD both significant enriched in GSE60993 and GSE66360 which included Lipid and atherosclerosis (ID: hsa05417), Neutrophil extracellular trap formation (ID: hsa04613), Fluid shear stress and atherosclerosis (ID: hsa05418) and MAPK signaling pathway (ID: hsa04010). In addition, most of these pathways were also validated by GSEA (Fig. 3D-F).

### Integration of PPI network and crucial module analysis

**Fig. 4:**
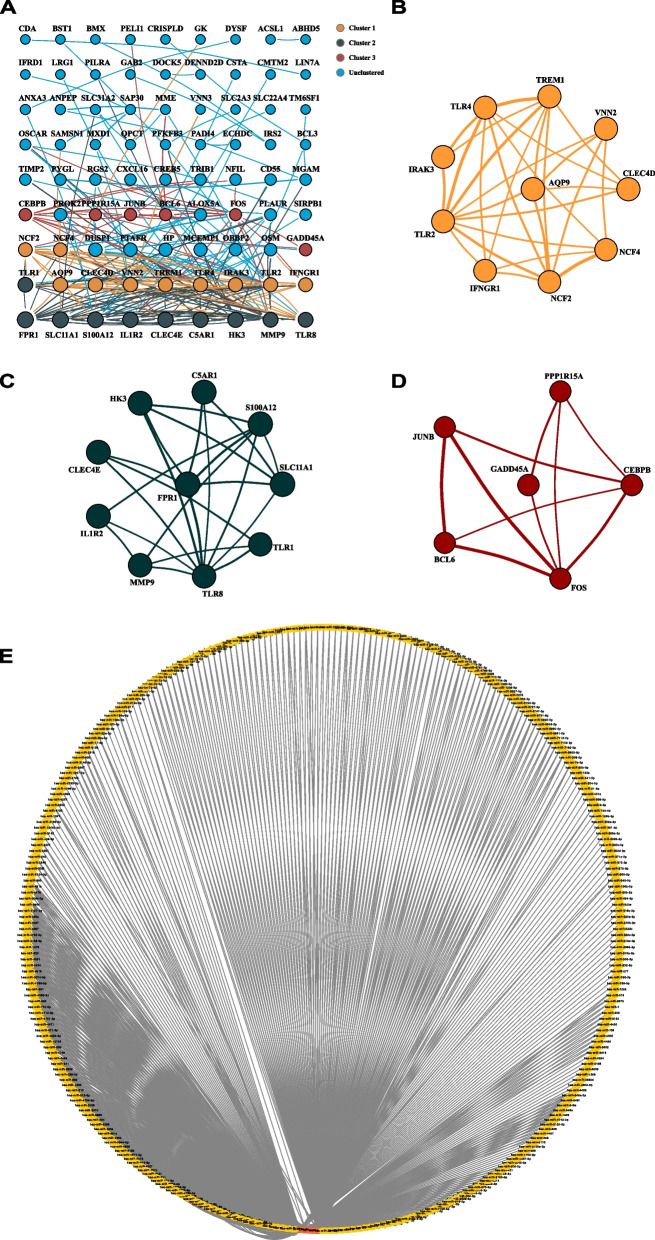
Analysis PPI networks of DEGs. **A** PPI network of DEGs. **B** Module 1 contained 10 nodes and 28 edges, MCODE score = 6.222. **C** Module 2 contained 10 nodes and 23 edges, MCODE score = 5.111. **D** Module 3 contained 6 nodes and 10 edges, MCODE score = 4.000. (The size of nodes represents the level of MCODE score and the width of line represents the level of combined score). **E** The network of reverse predicted miRNAs by 4 hub genes. And the highlight red color nodes represent 4 hub genes

We construct a PPI network for 113 overlapped DEGs, consisting of 113 nodes and 227 edges by using the STRING online database with a combined score > 0.4. Cytoscape software analyzes and shows this network (Fig. 4A). To better analyze the significance of DEGs in the PPI sub-networks, the cluster of the PPI network was identified and analyzed through MCODE (Degree Cutoff: 2, Node Score Cutoff: 0.2, K-Core: 2, Max Depth: 100). We obtained the three significant network modules with the score > 4: the first module with 10 nodes (MCODE Score = 6.222), the second module with 10 nodes (MCODE Score = 5.111) and the last module with 6 nodes (MCODE Score = 4). (Fig. 4B-D). Then, the CytoHubba plug-in revealed that the top 10 genes (TLR2, TLR4, TREM1, S100A12, MMP9, AQP9, CLEC4D, VNN2, SLC11A1, and FPR1) by the degree algorithm. And the genes with both the highest MCODE scores and the highest ranks of degrees were selected as hub genes. Based on the above conditions, we identified 10 hub genes (TLR2, TLR4, TREM1, S100A12, MMP9, AQP9, FOS, VNN2, SLC11A1, and FPR1) for subsequent analysis and validation. These genes were presented in Table S2.

### Hub genes screening, survival analysis, and expression validation

**Fig. 5:**
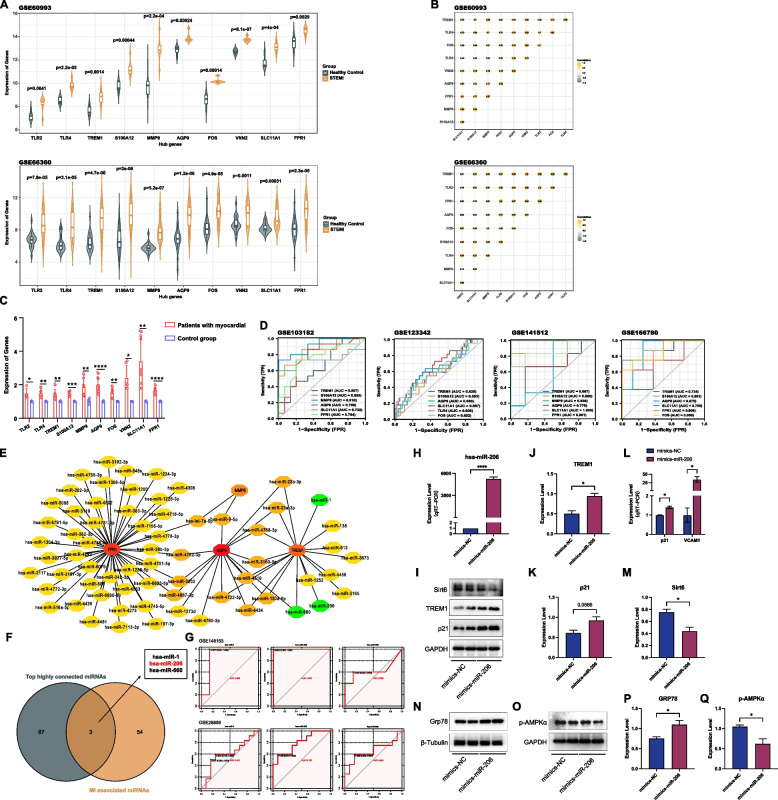
The prediction value in STEMI of hub genes as biomarkers and miR-206 may induce ECs senescence and endoplasmic reticulum stress. **A** The expression of hub genes. Compared with the control samples, the ten hub genes were up-regulated in MI samples in GSE60993 and GSE66360 respectively. **B** Heatmap analysis revealed similar correlation of these ten hub genes between MI samples and control. (Gold and gray denoted positive and negative correlation, respectively). **C** The level of 10 hub genes expression detected using RT-qPCR (****P < 0.0001, ***P < 0.001, **P < 0.01, *P < 0.05). **D** The ROC analysis of the hub genes in other several datasets about MI (Genes with an AUC value of > 0.50 in the four datasets are shown). **E** The top 70 highly connected miRNAs and mRNA identified by the CytoHubba plugin. (The highlight green nodes represent the MI associated miRNAs in the network; Other various degrees of color represent different ranking nodes of MCC method in CytoHubba plugin.) **F** The intersection of top highly connected miRNAs with the MI associate miRNAs. **G** The ROC analysis of the 3 candidate miRNAs in two GEO datasets. **H** qRT-PCR analysis of the levels of hsa-miR-206 in HUVECs after transfection of mimics-NC or mimics-miR-206. **I** The results of western blot analysis. **J** Western blot analysis of TREM1 in HUVECs after transfection of mimics-NC or mimics-miR-206. **H** Western blot analysis of p21 in HUVECs after transfection of mimics-NC or mimics-miR-206. **L** qRT-PCR analysis of the levels of p21 and VCAM1 in HUVECs after transfection of mimics-NC or mimics-miR-206. **M** Western blot analysis of Sirt6 in HUVECs after transfection of mimics-NC or mimics-miR-206. **N**-**O** The results of western blot analysis. **P** Western blot analysis of GRP78 in HUVECs after transfection of mimics-NC or mimics-miR-206. **Q** Western blot analysis of p-AMPKα in HUVECs after transfection of mimics-NC or mimics-miR-206. Uncropped protein Gels are provided in the Supplementary file (Supplementary Figs. 2 and 3). Data are presented as the mean ± SD. (****P < 0.0001, ***P < 0.001, **P < 0.01, *P < 0.05)

To evaluate the predictive value of these hub genes as biomarkers in STEMI. First and foremost, we observed that 10 DEGs had increased expression in both datasets (GSE60993 and GSE66360, Fig. 5A). Later, the expression of hub genes was performed by adopting Pearson's correlation analysis, and the results showed that there was a positive correlation between the genes’ expressions (Fig. 5B). To validate the putative value of hub genes as biomarkers for diagnosis or to monitor treatment efficacy in clinical translation. We extracted RNA from PBMCs derived from patients with STEMI to perform qPCR, while patients without STEMI were used as the normal control. And the results showed that the 10 hub genes were all elevated in the peripheral blood of STEMI patients, especially AQP9, FPR1, and S100A12 (Fig. 5C). On basis of above validation, at last, we analyzed ROC curves of prediction model and the AUC were listed in Fig. 5D. Therefore, combining KEGG pathway enrichment analysis, expression level validation, and ROC analysis, we finally selected four hub genes (AQP9, MMP9, FPR1, and TREM1) for subsequent analysis.

### Prediction and validation of hub miRNAs related to the progression of MI

In addition, miRNAs may have high prognostic value to evaluate the incidence and progression of MI because they can be easily detected in human serum and plasma. Thus, we reverse predicted the upstream miRNAs of the above four hub genes with predictive value. We summarize the predicted results of two databases (miRTarBase and starBase) and import them into the Cytoscape software. Subsequently, Cytohubba plugin revealed the highly connected miRNAs (Fig. 4E and Fig. 5E). MI associated miRNAs (hsa-miR-1, hsa-miR-206, and has-miR-660) were identified based on the HMDD database (Fig. 5F). To further evaluate the prognostic value of the 3 miRNAs and their clinical applications, we also performed AUC analysis on a MI-related dataset (GSE148153, Fig. 5G) and an AS-related dataset (GSE28858, Fig. 5G) of the GEO database. And we selected the best miRNA (miR-206) with the greatest AUC values.

### MiR-206 may induce ECs senescence and endoplasmic reticulum stress

Thus, next we engaged in the follow-up analyses to miR-206. After overexpression of miR-206, we explored the molecular mechanism of its regulation of MI in a cell model. Firstly, qRT-PCR results showed that miR-206 expression was significantly increased in the transfected mimics group compared to the NC group (Fig. 5H), indicating that miR-206 was successfully overexpressed in HUVECs. ECs senescence is engaged in the process of AS and thus assists in the development of MI [36]. The western blot analysis revealed that overexpressing of miR-206 led to an upregulation of senescent marker p21, whereas the expression of senescence protective factor Sirt6 was reduced (Fig. 5I, J,K and M). Similarly, the qPCR results showed that miR-206 overexpression upregulated the expression of p21 and inflammatory factor VCAM1 (Fig. 5L). Endoplasmic reticulum (ER) stress is involved in a series of physiological processes before and after the development of MI [37]. Therefore, we observed the expression levels of ER stress chaperone protein glucose regulatory protein 78 (GRP78) in HUVECs after overexpression of miR-206 (Fig. 5N and P). Correspondingly, we found a decrease in the phosphorylation and activation of AMP-activated protein kinase (AMPK) (Fig. 5O and Q). Given this evidence, we believe that miR-206 may contribute to the occurrence of MI by promoting ECs senescence and ER stress, and the AMPK pathway is one of the potential mechanisms. This corresponds to the results of KEGG pathway enrichment analysis.

### MiR-206 induces mitochondrial ROS production via TREM1

**Fig. 6:**
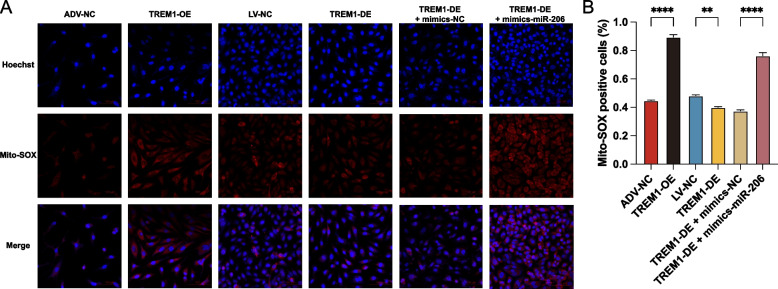
MiR-206 induces mitochondrial ROS production via TREM1. **A** The mitochondrial ROS levels in HUVECs after transfection was detected by MitoSOX Red fluorogenic dye and images were taken with Nikon Confocal laser-scanning microscopy. And quantification of per image was calculated by Image J software and plotted using GraphPrism. Data are presented as the mean ± SD. (****P < 0.0001, ***P < 0.001, **P < 0.01, *P < 0.05)

To clarify the regulatory effect of miR-206 on TREM1, we explored the expression level of TREM1 during overexpression of miR-206. The WB results in Fig. 5I and J demonstrate that the level of TREM1 was upregulated following the overexpression of miR-206. High mitochondrial ROS levels can lead to various biological dysfunctions, including oxidative stress, the accumulation of mtDNA mutations, and even ER stress [38, 39]. Mitochondrial ROS in HUVECs was detected with MitoSOX™ Red reagent. The confocal microscopy suggested that mitochondrial ROS levels were significantly elevated in the TREM1 overexpression group compared to the control group, whereas the converse result was obtained in the silenced TREM1 group. Moreover, we also observed a higher level of mitochondrial ROS in miR-206 overexpression and TREM1 silencing group. Taken together, our results showed that miR-206 could increase the production of mitochondrial ROS via TREM1 and then contribute the occurrence of ECs senescence and ER stress (Fig. 6).

## Discussion

MI has been associated with sudden cardiac death and cardiac dysfunction, especially STEMI, which exhibits higher mortality rates. In recent years, despite the progress of PCI, antithrombotic therapy, and anticoagulation, the prognosis for STEMI is still not ideal. Therefore, it is necessary to identify novel and specific biomarkers to facilitate early diagnosis, aiming to implement more effective interventions to improve patient outcomes.

In this study, consequently, we performed an in-depth analysis of the microarray expression profile from the GSE60993 and GSE66360 datasets. In GSE60993, we discovered a total of 642 DEGs (435 up, 207 down), while in GSE66360, we discovered 608 DEGs (485 up, 123 down). Subsequently, we obtained 113 overlapped DEGs from the two datasets and identified the top 10 hub genes (TLR2, TLR4, TREM1, S100A12, MMP9, AQP9, FOS, VNN2, SLC11A1, FPR1) from the 113 DEGs by constructing PPI network using Cytoscape software. We executed GO enrichment and KEGG pathway analysis, as well as subsequent PPI network construction and GSEA analysis, to gain insight into the role of DEGs in biological processes and associated molecular pathways. To further analyze the expression levels of hub genes from an individual database. The mRNA expression levels of 10 hub genes were verified in GSE60993 and GSE66360 taken separately. Subsequently, analysis of correlation was performed for the 10 hub genes, and the results show that FPR1 and AQP9 had the highest positive correlations, and MMP9 and S100A12 had higher positive correlations. We also detected changes in the expression levels of hub genes in the PBMC of STEMI patients using qRT-PCR separately, patients without MI were used as controls. Concurrently, analysis of ROC curves in different GEO databases (GSE103182, GSE123342, GSE141512, and GSE166780) indicates MMP9, S100A12, FPR1, and AQP9 have a good predictive ability for MI especially STEMI. At last, we reverse predicted the upstream miRNA from the four hub genes and then selected miR-206 and TREM1 for subsequent analysis.

Following analysis of GO enrichment and KEGG pathways for identified DEGs, the most significant p value enriched terms consist of neutrophil activation, T cell activation, regulation of innate immune response, and regulation of mononuclear cell proliferation. Among others, neutrophil activation plays a crucial role in myocardial injury following acute MI. It damages cardiomyocytes through the generation of high amounts of reactive oxygen species (ROS) and the release of proteolytic enzymes that can exacerbate local tissue injury [40, 41]. Furthermore, innate immune response regulation contributes to the progression of complications after the MI incident [42, 43]. It will trigger the activation of immune cells (T cell) and cause the adaptive immune response after the production of inflammatory factors (TGF-α, IL-1 and IL-6) [44]. The process aims to generate anti-inflammatory factors (IL-10) that resist the above inflammatory process [45].

On the other hands, KEGG pathway annotation analysis indicated that chiefly participated in MAPK signaling pathway, Lipid and atherosclerosis, Neutrophil extracellular trap formation, Cytokine-cytokine receptor interaction, TNF signaling pathway and NF-kappa B pathway. Several studies have indicated that MAPK signaling cascades’ response plays an important role in oxidative stress-induced apoptosis [46, 47]. The downstream targets (ERK1/2, p38, and JNK) of the MAPK pathway are activated following MI [48, 49]. whereas ERK activation promotes cell proliferation and differentiation, and p38/JNK mediates apoptosis and the release of pro-inflammatory factors [50].

MiRNA is a class of endogenous non-coding RNA composed of 20–25 nucleotides. And sometimes miRNA regulates gene expression by promoting their degradation [51]. MiR-206 is widely distributed and expressed in various tissues and cell types, including brain [52], and cancer cell lines [53]. It mediates diverse functions, such as tissue growth and differentiation [52], angiogenesis inhibition [54], and tumor suppression [53]. MiR-206 has also been shown to be a crucial factor for skeletal muscle and cardiac development and function [55]. Several studies have found that miR-206 is always expressed in the cardiovascular system and may be associated with CVD. The expression of miR-206 was markedly downregulated in the heart tissue of rats with myocardial ischemia–reperfusion injury, and the overexpression of lncRNA MALAT1 significantly suppressed miR-206 expression [56]. Another study found miR-206 mediates ox-LDL induced ECs injury [57]. On the other hand, miR-206 can also delay the progression of AS by inhibiting the proliferation of vascular smooth muscle cells [58]. Moreover, miR-206 has been implicated in various cardiovascular conditions associated with mitochondrial dysfunction, such as ischemia–reperfusion injury [59], and diabetic cardiomyopathy [60]. For instance, miR-206 can mediate YAP-induced cardiac hypertrophy and survival [61]. However, none of the above studies have revealed whether miR-206 affects the progression of cardiovascular diseases by regulating mitochondrial function. Also, the role of miR-206 in endothelial cells and the regulation of mitochondrial function remains to be further elucidated.

In addition, the expression trends of miR-206 are also consistent with the construction principles of the ceRNA network, which lays the foundation for further analysis. And we found that miR-206 promotes ECs senescence and ER stress upon overexpression. There is strong evidence that the pathophysiology of human AS is influenced by ECs senescence and ER stress [62, 63]. Our findings may reveal an important role for miR-206 in inducing ECs senescence and ER stress and provide a fresh perspective on miRNA in the progression of AS and MI. In addition, according to earlier research, AMPK phosphorylation and activation are associated with ER stress [64] and cell senescence [65]. We found that the phosphorylation and activation of AMPK were reduced when miR-206 was overexpressed.

In addition, the findings of this study indicated that the four hub genes (AQP9, MMP9, FPR1, and TREM1) may serve as potential CVD biomarkers for MI, particularly STEMI. AQP9 belongs to aquaporins (AQPs), which further take part in maintain the expression of MMP9 appears to increase in human atherosclerotic plaques [66]. It indicated that MMP9 may predict the happen of CVD included MI. FPR1, which belongs to the formyl peptide receptors (FPRs), is one of the most intensively studied classes of the G-protein-coupled receptors (GPCRs) [67]. FPR1 was found to express in the tissues and cells associated with inflammation, which will cause a series of ischemia reperfusion (IR) inflammatory reactions when FPR1 is activated [68]. The other family member of FPR1, FPR2, contributes to angiogenesis in the repair of the myocardium post-MI [69]. In our study, FPR1 was found to play a significant role in the PPI network, and its expression level was confirmed by integrating the analysis of qRT-PCR and the ROC curve, which further indicated its prediction value in the STEMI.

TREM1 is an immunological receptor that derives from neutrophils, macrophages, and mature monocytes and works as an immune response amplifier in a variety of inflammatory diseases [70]. In a previous investigation, the plasma concentration of patients with acute MI was shown to contain the soluble form of TREM-1 (sTREM-1), which was also employed as an independent predictor of death [71]. It also indicated that Plasma TREM1 level may as the biomarker to predict major adverse cardiovascular event (MACE) in patients with stable CVD concurrently. In this study, we further provided evidence that miR-206 may up-regulate mitochondrial ROS accumulation via increased the expression of TREM1. Iurlaro et al. study demonstrated that excessive ROS levels in cells induce ER stress, which in turn increases GRP78 protein to promote early apoptosis [39]. Additionally, ROS are considered to be a significant role in cellular senescence [72]. The cumulative effect of ROS leads to senescence and is a major factor in CVD [36].

Of course, our study has some limitations. Firstly, relatively few the GEO datasets numbers and the sample size of the dataset used in this study. Moreover, although we have found some crucial DEGs and enriched KEGG pathways, the potential mechanisms of the abnormal effects of these pathways on MI have not yet been fully elucidated. At last, although we revealed that miR-206 induces ECs senescence and ER stress by upregulating mitochondrial ROS levels via TREM1, the specific mechanisms by which miR-206 regulates ECs senescence and ER stress through the AMPK pathway still need to be further explored.

## Conclusions

Altogether, we screened out 642 DEGs from GSE60993 dataset and 608 DEGs from GSE66360 dataset respectively by a series of bioinformatic analysis. Then we identified four of the top 10 hub genes, including AQP9, MMP9, FPR1, and TREM1, that might play a crucial role in the progression of MI through experiments.

Finally, we selected miR-206 based on a reverse mRNA prediction model by integrating genomics and various bioinformatics analyses. Finally, we found miR-206 may induce ECs senescence and ER stress by upregulating mitochondrial ROS levels via TREM1, which can deepen our understanding of the occurrence and progression of MI. Thus, our results provide unambiguous evidence for shed light on molecular mechanisms and treatment targets exploration of MI.

### Supplementary Information

**Additional file 1.** **Table S1.** qPCR primers used in this study.

**Additional file 2.** **Table S2.** The information of 10 hub genes identified in this study.

**Additional file 3:** **Supplementary Figure 1.** PCA plot of samples in different groups. **Supplementary Figure 2.** The original Gels of Fig. 5I were presented. **Supplementary Figure 3.** The original Gels of Fig. 5N and O were presented.

## Data Availability

The datasets generated and analysed during the current study are available in the Gene Expression Omnibus (GEO, https://www.ncbi.nlm.nih.gov/geo/) repository, [GSE66360, GSE60993, GSE103182, GSE123342, GSE141512, GSE166780, GSE148153, and GSE28858].

## References

[1] Zhao D, Liu J, Wang M (2019). Epidemiology of cardiovascular disease in China: current features and implications [J]. Nat Rev Cardiol.

[2] Roger VL (2007). Epidemiology of myocardial infarction [J]. Med Clin North Am.

[3] Hedayati T, Yadav N, Khanagavi J (2018). Non-ST-segment acute coronary syndromes [J]. Cardiol Clin.

[4] Nakajima T, Yamazaki K (2009). Periodontal disease and risk of atherosclerotic coronary heart disease [J]. Odontology.

[5] De Luca G, Suryapranata H, Ottervanger JP (2004). Time delay to treatment and mortality in primary angioplasty for acute myocardial infarction: every minute of delay counts [J]. Circulation.

[6] Yusuf S, Reddy S, Ounpuu S (2001). Global burden of cardiovascular diseases: part I: general considerations, the epidemiologic transition, risk factors, and impact of urbanization [J]. Circulation.

[7] Gustafsson AB, Gottlieb RA (2008). Heart mitochondria: gates of life and death [J]. Cardiovasc Res.

[8] Hortmann M, Robinson S, Mohr M (2017). Circulating HtrA2 as a novel biomarker for mitochondrial induced cardiomyocyte apoptosis and ischemia-reperfusion injury in ST-segment elevation myocardial infarction [J]. Int J Cardiol.

[9] Li G, Hu R, Guo Y (2019). Circulating sulfatide, a novel biomarker for ST-segment elevation myocardial infarction [J]. J Atheroscler Thromb.

[10] Jf OS, Neylon A, Mcgorrian C (2016). miRNA-93-5p and other miRNAs as predictors of coronary artery disease and STEMI [J]. Int J Cardiol.

[11] Bochaton T, Paccalet A, Jeantet P (2020). Heat shock protein 70 as a biomarker of clinical outcomes after STEMI [J]. J Am Coll Cardiol.

[12] Frydland M, Ostrowski SR, Møller JE (2018). Plasma concentration of biomarkers reflecting endothelial cell- and glycocalyx damage are increased in patients with suspected ST-elevation myocardial infarction complicated by cardiogenic shock [J]. Shock.

[13] Erhard F, Haas J, Lieber D (2014). Widespread context dependency of microRNA-mediated regulation [J]. Genome Res.

[14] Guo Z, Maki M, Ding R (2014). Genome-wide survey of tissue-specific microRNA and transcription factor regulatory networks in 12 tissues [J]. Sci Rep.

[15] Romaine SP, Tomaszewski M, Condorelli G (2015). MicroRNAs in cardiovascular disease: an introduction for clinicians [J]. Heart.

[16] Wang C, Jing Q (2018). Non-coding RNAs as biomarkers for acute myocardial infarction [J]. Acta Pharmacol Sin.

[17] Huang Y, Yang Y, He Y (2016). MicroRNA-208a potentiates angiotensin ii-triggered cardiac myoblasts apoptosis via inhibiting Nemo-like Kinase (NLK) [J]. Curr Pharm Des.

[18] Tony H, Meng K, Wu B (2015). MicroRNA-208a dysregulates apoptosis genes expression and promotes cardiomyocyte apoptosis during ischemia and its silencing improves cardiac function after myocardial infarction [J]. Mediators Inflamm.

[19] Davis S, Meltzer PS (2007). GEOquery: a bridge between the Gene Expression Omnibus (GEO) and BioConductor [J]. Bioinformatics.

[20] Ritchie ME, Phipson B, Wu D (2015). limma powers differential expression analyses for RNA-sequencing and microarray studies [J]. Nucleic Acids Res.

[21] Gene Ontology Consortium: going forward [J]. Nucleic Acids Res, 2015, 43(Database issue): D1049–56. 10.1093/nar/gku1179 PMC4383973 25428369

[22] Kanehisa M, Furumichi M, Tanabe M (2017). KEGG: new perspectives on genomes, pathways, diseases and drugs [J]. Nucleic Acids Res.

[23] Subramanian A, Tamayo P, Mootha VK (2005). Gene set enrichment analysis: a knowledge-based approach for interpreting genome-wide expression profiles [J]. Proc Natl Acad Sci U S A.

[24] Yu G, Wang LG, Han Y (2012). clusterProfiler: an R package for comparing biological themes among gene clusters [J]. OMICS.

[25] Walter W, Sánchez-Cabo F, Ricote M (2015). GOplot: an R package for visually combining expression data with functional analysis [J]. Bioinformatics.

[26] Stelzl U, Worm U, Lalowski M (2005). A human protein-protein interaction network: a resource for annotating the proteome [J]. Cell.

[27] Szklarczyk D, Gable AL, Nastou KC (2021). The STRING database in 2021: customizable protein-protein networks, and functional characterization of user-uploaded gene/measurement sets [J]. Nucleic Acids Res.

[28] Shannon P, Markiel A, Ozier O (2003). Cytoscape: a software environment for integrated models of biomolecular interaction networks [J]. Genome Res.

[29] Bader GD, HoguE CW (2003). An automated method for finding molecular complexes in large protein interaction networks [J]. BMC Bioinformatics.

[30] Chin CH, Chen SH, Wu HH (2014). cytoHubba: identifying hub objects and sub-networks from complex interactome [J]. BMC Syst Biol.

[31] Thygesen K, Alpert JS, Jaffe AS (2018). Fourth universal definition of myocardial infarction (2018) [J]. Circulation.

[32] Huang HY, LiN YC, Li J (2020). miRTarBase 2020: updates to the experimentally validated microRNA-target interaction database [J]. Nucleic Acids Res.

[33] Li JH, Liu S, Zhou H (2014). decoding miRNA-ceRNA, miRNA-ncRNA and protein-RNA interaction networks from large-scale CLIP-Seq data [J]. Nucleic Acids Res.

[34] Huang Z, Shi J, Gao Y (2019). HMDD v3.0: a database for experimentally supported human microRNA-disease associations [J]. Nucleic Acids Res.

[35] Tang Y, Horikoshi M, Li WX (2016). ggfortify: unified interface to visualize statistical results of popular R packages [J]. R JOURNAL.

[36] Hu C, Zhang X, Teng T (2022). Cellular senescence in cardiovascular diseases: a systematic review [J]. Aging Dis.

[37] Li S, Ma J, Li JB (2018). Over-expression of calpastatin attenuates myocardial injury following myocardial infarction by inhibiting endoplasmic reticulum stress [J]. J Thorac Dis.

[38] Marchi S, Giorgi C, Suski JM (2012). Mitochondria-ros crosstalk in the control of cell death and aging [J]. J Signal Transduct.

[39] Iurlaro R, Muñoz-Pinedo C (2016). Cell death induced by endoplasmic reticulum stress [J]. Febs j.

[40] Timmers L, Pasterkamp G, De Hoog VC (2012). The innate immune response in reperfused myocardium [J]. Cardiovasc Res.

[41] Bonaventura A, Montecucco F, Dallegri F (2016). Cellular recruitment in myocardial ischaemia/reperfusion injury [J]. Eur J Clin Invest.

[42] Epelman S, Liu PP, Mann DL (2015). Role of innate and adaptive immune mechanisms in cardiac injury and repair [J]. Nat Rev Immunol.

[43] Swirski FK, Nahrendorf M (2013). Leukocyte behavior in atherosclerosis, myocardial infarction, and heart failure [J]. Science.

[44] Kaur K, Sharma AK, Singal PK (2006). Significance of changes in TNF-alpha and IL-10 levels in the progression of heart failure subsequent to myocardial infarction [J]. Am J Physiol Heart Circ Physiol.

[45] Frangogiannis NG, Mendoza LH, Lindsey ML (2000). IL-10 is induced in the reperfused myocardium and may modulate the reaction to injury [J]. J Immunol.

[46] Liu J, Mao W, Ding B (2008). ERKs/p53 signal transduction pathway is involved in doxorubicin-induced apoptosis in H9c2 cells and cardiomyocytes [J]. Am J Physiol Heart Circ Physiol.

[47] Hsieh CC, Papaconstantinou J (2006). Thioredoxin-ASK1 complex levels regulate ROS-mediated p38 MAPK pathway activity in livers of aged and long-lived Snell dwarf mice [J]. Faseb j.

[48] Engelbrecht AM, Engelbrecht P, Genade S (2005). Long-chain polyunsaturated fatty acids protect the heart against ischemia/reperfusion-induced injury via a MAPK dependent pathway [J]. J Mol Cell Cardiol.

[49] Li DY, Tao L, Liu H (2006). Role of ERK1/2 in the anti-apoptotic and cardioprotective effects of nitric oxide after myocardial ischemia and reperfusion [J]. Apoptosis.

[50] Shimada K, Nakamura M, Ishida E (2003). Roles of p38- and c-jun NH2-terminal kinase-mediated pathways in 2-methoxyestradiol-induced p53 induction and apoptosis [J]. Carcinogenesis.

[51] Liu J, Jia Y, Jia L (2019). MicroRNA 615–3p inhibits the tumor growth and metastasis of NSCLC via inhibiting IGF2 [J]. Oncol Res.

[52] Lee ST, Chu K, Jung KH (2012). miR-206 regulates brain-derived neurotrophic factor in Alzheimer disease model [J]. Ann Neurol.

[53] Nohata N, Hanazawa T, Enokida H (2012). microRNA-1/133a and microRNA-206/133b clusters: dysregulation and functional roles in human cancers [J]. Oncotarget.

[54] Lin CY, Lee HC, Fu CY (2013). MiR-1 and miR-206 target different genes to have opposing roles during angiogenesis in zebrafish embryos [J]. Nat Commun.

[55] Townley-Tilson WH, Callis TE, Wang D (2010). MicroRNAs 1, 133, and 206: critical factors of skeletal and cardiac muscle development, function, and disease [J]. Int J Biochem Cell Biol.

[56] Jing H, Wang C, Zhao L (2021). Propofol protects cardiomyocytes from hypoxia/reoxygenation injury via regulating MALAT1/miR-206/ATG3 axis [J]. J Biochem Mol Toxicol.

[57] Gao Y, Yue J, Huang Z (2021). LncRNA MIAT Mediates ox-LDL-Induced Endothelial Cell Injury Via miR-206/RAB22A Axis [J]. J Surg Res.

[58] Xing T, Du L, Zhuang X (2017). Upregulation of microRNA-206 induces apoptosis of vascular smooth muscle cells and decreases risk of atherosclerosis through modulating FOXP1 [J]. Exp Ther Med.

[59] Zhai C, Qian Q, Tang G (2017). MicroRNA-206 Protects against Myocardial Ischaemia-Reperfusion Injury in Rats by Targeting Gadd45β [J]. Mol Cells.

[60] Delfan M, Amadeh Juybari R, Gorgani-Firuzjaee S (2022). High-intensity interval training improves cardiac function by miR-206 dependent HSP60 induction in diabetic rats [J]. Front Cardiovasc Med.

[61] Yang Y, Re DPD, Nakano N (2015). miR-206 mediates YAP-induced cardiac hypertrophy and survival [J]. Circ Res.

[62] Minamino T, Komuro I (2007). Vascular cell senescence: contribution to atherosclerosis [J]. Circ Res.

[63] Yang S, Wu M, Li X (2020). Role of endoplasmic reticulum stress in atherosclerosis and its potential as a therapeutic target [J]. Oxid Med Cell Longev.

[64] Luo H, Lan C, Fan C (2022). Down-regulation of AMPK/PPARδ signalling promotes endoplasmic reticulum stress-induced endothelial dysfunction in adult rat offspring exposed to maternal diabetes [J]. Cardiovasc Res.

[65] Wang Z, Chen Z, Jiang Z (2019). Cordycepin prevents radiation ulcer by inhibiting cell senescence via NRF2 and AMPK in rodents [J]. Nat Commun.

[66] Galis ZS, Sukhova GK, Lark MW (1994). Increased expression of matrix metalloproteinases and matrix degrading activity in vulnerable regions of human atherosclerotic plaques [J]. J Clin Invest.

[67] Schiffmann E, Corcoran BA, Wahl SM (1975). N-formylmethionyl peptides as chemoattractants for leucocytes [J]. Proc Natl Acad Sci U S A.

[68] Gavins FN (2010). Are formyl peptide receptors novel targets for therapeutic intervention in ischaemia-reperfusion injury? [J]. Trends Pharmacol Sci.

[69] Heo SC, Kwon YW, Jang IH (2017). Formyl peptide receptor 2 is involved in cardiac repair after myocardial infarction through mobilization of circulating angiogenic cells [J]. Stem Cells.

[70] Bouchon A, Facchetti F, Weigand MA (2001). TREM-1 amplifies inflammation and is a crucial mediator of septic shock [J]. Nature.

[71] Boufenzer A, Lemarié J, Simon T (2015). TREM-1 mediates inflammatory injury and cardiac remodeling following myocardial infarction [J]. Circ Res.

[72] Davalli P, Mitic T, Caporali A (2016). ROS, Cell Senescence, and novel molecular mechanisms in aging and age-related diseases [J]. Oxid Med Cell Longev.

