# Supplementary material for: MiR-206 may regulate mitochondrial ROS contribute to the progression of Myocardial infarction via TREM1
Source: BMC Cardiovasc Disord. 2023 Sep 20;23:470. doi: 10.1186/s12872-023-03481-8 (PMC10512505; doi:10.1186/s12872-023-03481-8)
Supplement: Supplementary file 1 — Additional file 1. Table S1. qPCR primers used in this study. [file 12872_2023_3481_MOESM1_ESM.docx]

Table S1. qPCR primers used in this study.

| **Name** | **Species** | **Primer sequence** |
| --- | --- | --- |
| *TLR2-F* | Human/mouse | TTATCCAGCACACGAATACACAG |
| *TLR2-R* | Human | AGGCATCTGGTAGAGTCATCAA |
| *TLR4-F* | Human | AGACCTGTCCCTGAACCCTAT |
| *TLR4-R* | Human | CGATGGACTTCTAAACCAGCCA |
| *TREM1-F* | Human | GAACTCCGAGCTGCAACTAAA |
| *TREM1-R* | Human | TCTAGCGTGTAGTCACATTTCAC |
| *S100A12-F* | Human | CTTACAAAGGAGCTTGCAAACA |
| *S100A12-R* | Human | TGGCTACCAGGGATATGAATTC |
| *MMP9-F* | Human | AGACCTGGGCAGATTCCAAAC |
| *MMP9-R* | Human | CGGCAAGTCTTCCGAGTAGT |
| *AQP9-F* | Human | TGTCTCTTTGGACGGATGAAATG |
| *AQP9-R* | Human | TCTCCCACGATCAGCAGTTTT |
| *FOS-F* | Human | GGGGCAAGGTGGAACAGTTAT |
| *FOS-R* | Human | CCGCTTGGAGTGTATCAGTCA |
| *VNN2-F* | Human | CAGGGTGCTCGAATCATTGTG |
| *VNN2-R* | Human | CACGGAATCCAGTTCACCTGA |
| *SLC11A1-F* | Human | CTTCAGCCTGCGGAAGCTAT |
| *SLC11A1-R* | Human | TCTGACTCGATGTTTCCTGGG |
| *FPR1-F* | Human | GCTCCTCACATTGCCAGTTAT |
| *FPR1-R* | Human | CGTTGGTCCAGGGCGAAAA |
| *Hsa_miR-206-F* | Human | GCGCGTGGAATGTAAGGAAGT |
| *Hsa_miR-206-R* | Human | AGTGCAGGGTCCGAGGTATT |
| *Hsa_miR-206-RT* | Human | GTCGTATCCAGTGCAGGGTCCG  AGGTATTCGCACTGGATACGAC |
| *GAPDH-F* | Human | AGAAGGCTGGGGCTCATTTG |
| *GAPDH-R* | Human | GCAGGAGGCATTGCTGATGAT |
| *β-actin-F* | Human | CTCCATCCTGGCCTCGCTGT |
| *β-actin-R* | Human | GCTGTCACCTTCACCGTTCC |
|  |  |  |
|  |  |  |
|  |  |  |
|  |  |  |
