# Supplementary material for: MiR-206 may regulate mitochondrial ROS contribute to the progression of Myocardial infarction via TREM1
Source: BMC Cardiovasc Disord. 2023 Sep 20;23:470. doi: 10.1186/s12872-023-03481-8 (PMC10512505; doi:10.1186/s12872-023-03481-8)
Supplement: Supplementary file 2 — Additional file 2. Table S2. The information of 10 hub genes identified in this study. [file 12872_2023_3481_MOESM2_ESM.docx]

**Table S2.** **The information of 10 hub genes identified in this study.**

| Degree Gene symbol Gene full name Function |
| --- |
| 26 TLR4 Toll like receptor 4 It plays a fundamental role in pathogen recognition and activation of innate immunity.  25 TLR2 Toll like receptor 2 It plays a fundamental role in pathogen recognition and activation of innate immunity.    23 S100A12 S100 calcium binding protein A12 It was widely expressed in leukocyte, and nduced cytokine secretion mediated by NF-kappa B pathway.  22 MMP9 Matrix metallopeptidase 9 It was important for angiogenesis, cell migration, growth and apoptosis.  21 FPR1 Formyl peptide receptor 1 It was participated in inflammation related myocardial infarction.  20 TREM1 Triggering receptor expressed on myeloid cells 1 This protein amplifies neutrophil and monocyte-mediated inflammatory responses.  18 SLC11A1 Solute carrier family 11 member 1 This gene encodes a cell surface  glycoprotein which is typically expressed on endothelial cells and regulates angiogenesis.  18 FOS Fos proto-oncogene The gene FOS proteins have been implicated as regulators of cell proliferation, differentiation, and transformation.  12 VNN2 Vanin 2 It may play a key role in oxidative-stress response..  11 AQP9 Aquaporin 9 It is of great importance in maintaining the water balance of cells. |
