# Supplementary material for: MiR-206 may regulate mitochondrial ROS contribute to the progression of Myocardial infarction via TREM1
Source: BMC Cardiovasc Disord. 2023 Sep 20;23:470. doi: 10.1186/s12872-023-03481-8 (PMC10512505; doi:10.1186/s12872-023-03481-8)
Supplement: Supplementary file 3 — Additional file 3: Supplementary Figure 1. PCA plot of samples in different groups. Supplementary Figure 2. The original Gels of Fig. 5I were presented. Supplementary Figure 3. The original Gels of Fig. 5N and O were presented. [file 12872_2023_3481_MOESM3_ESM.pdf]

Supplementary file

Supplementary Figure 1. PCA plot of samples in different groups.
Supplementary Figure 2. The original Gels of Fig. 5I were presented.
Supplementary Figure 3. The original Gels of Fig. 5N and Fig. 5O were presented.

Supplementary Figure 1

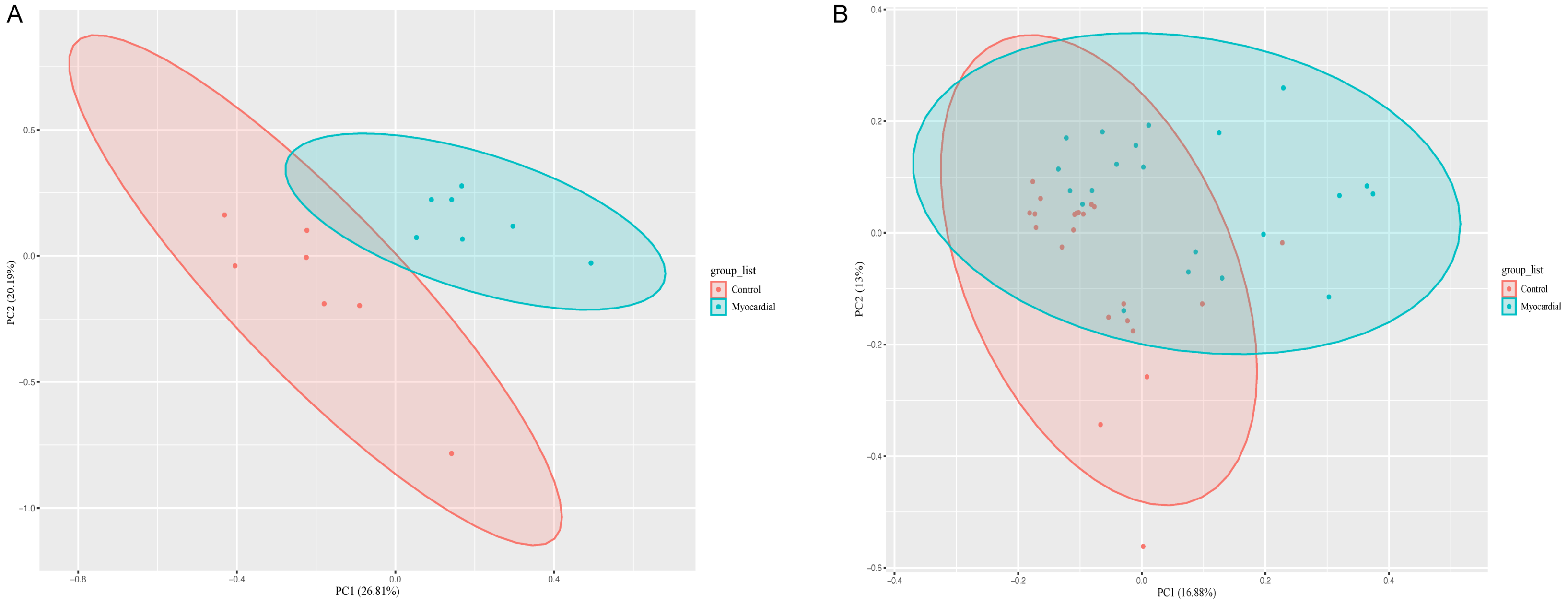

Supplementary Figure 1. PCA plot of samples in different groups.

Supplementary Figure 2

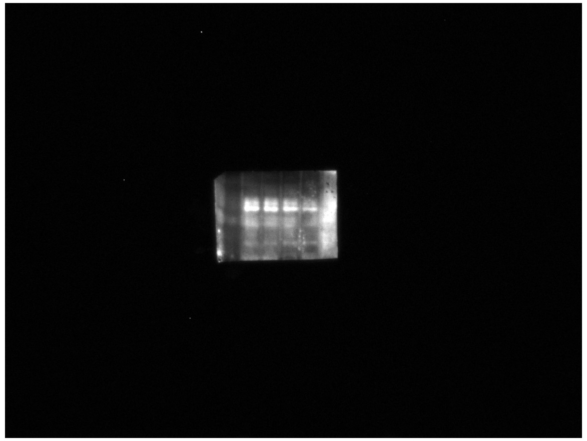

Sirt6
42KD

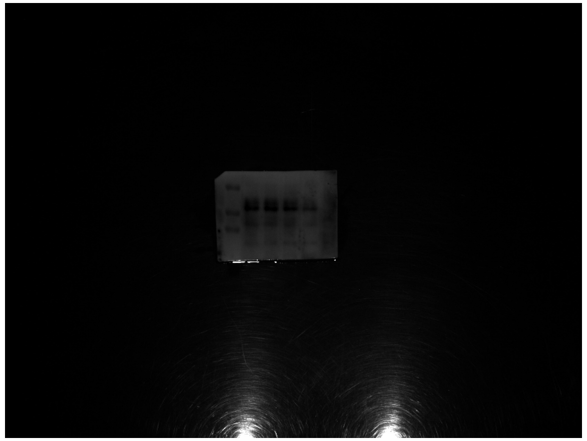

Sirt6 with marker version
42KD

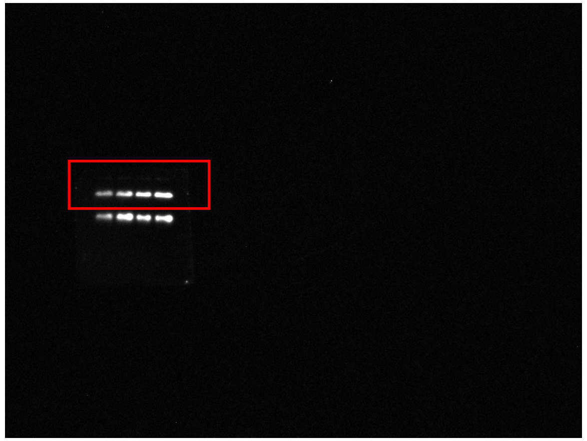

TREM1
26KD

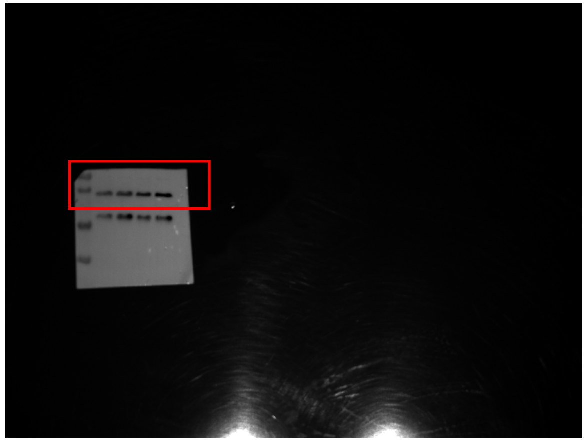

TREM1 with marker version
26KD

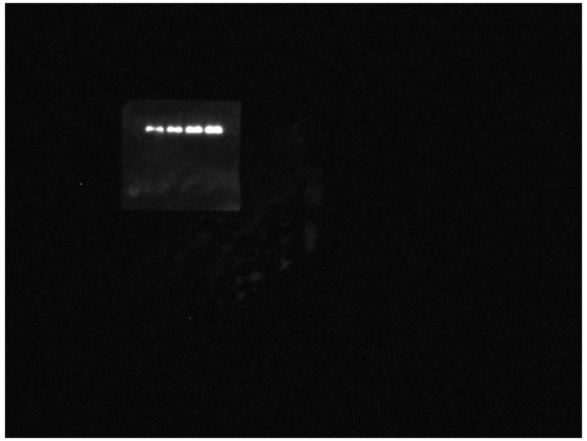

p21
21KD

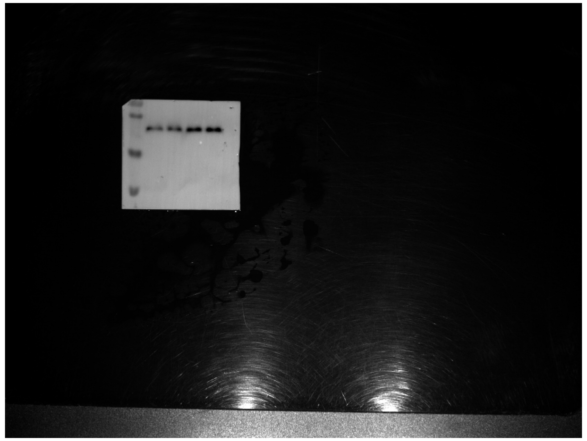

p21 with marker version
21KD

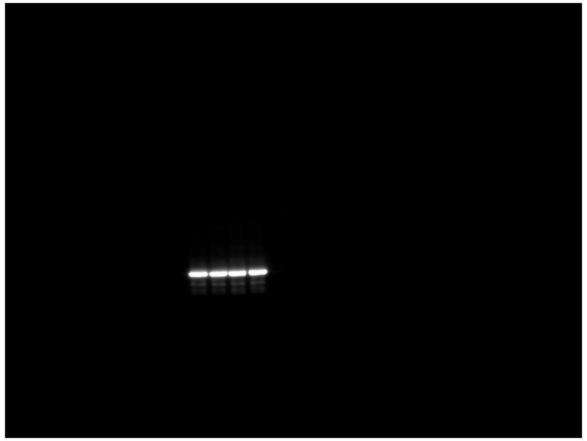

GAPDH
37KD

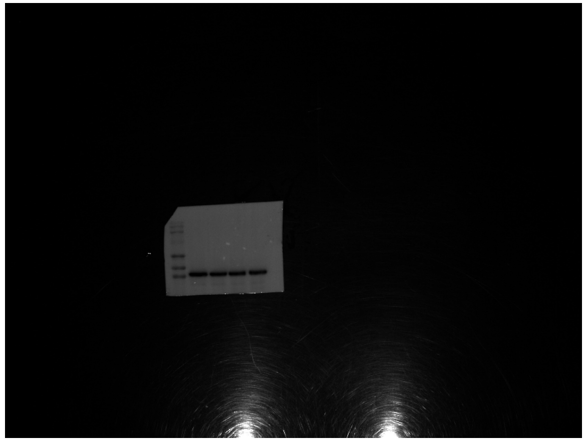

GAPDH with marker version
37KD

The original Gels of Fig. 5I were presented.

Supplementary Figure 3

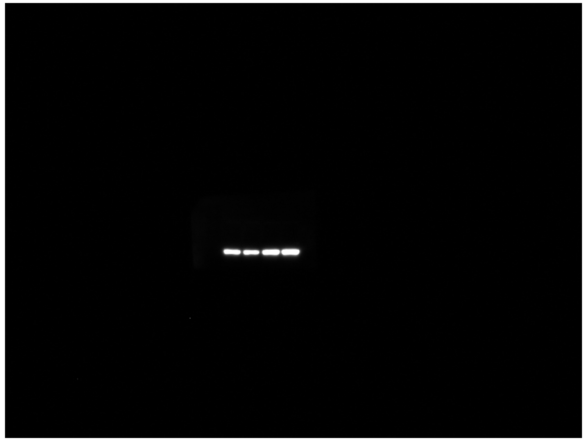
GRP78
78KD

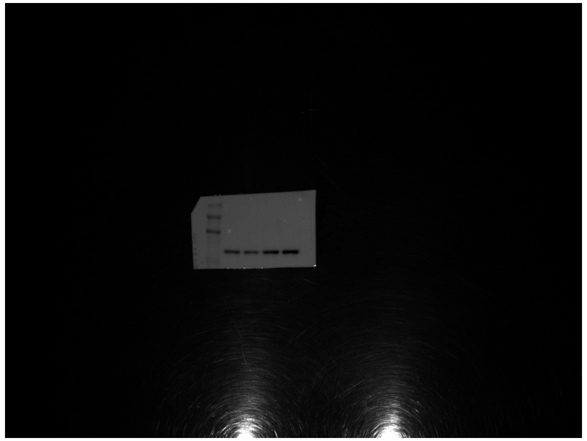
GRP78 with marker version
78KD

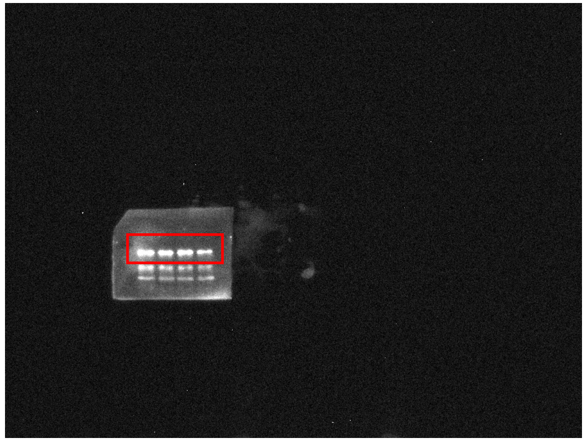
p-AMPKα
62KD

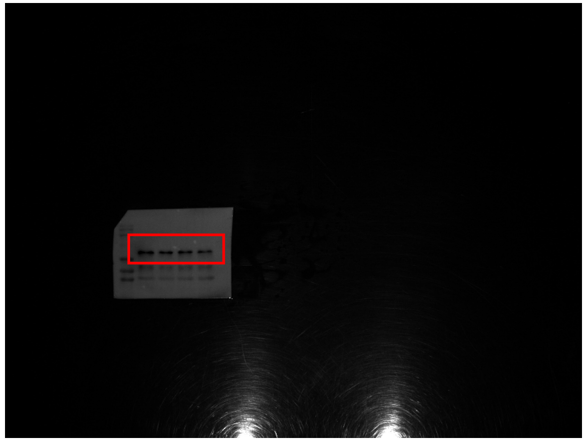
p-AMPKα with marker version
62KD

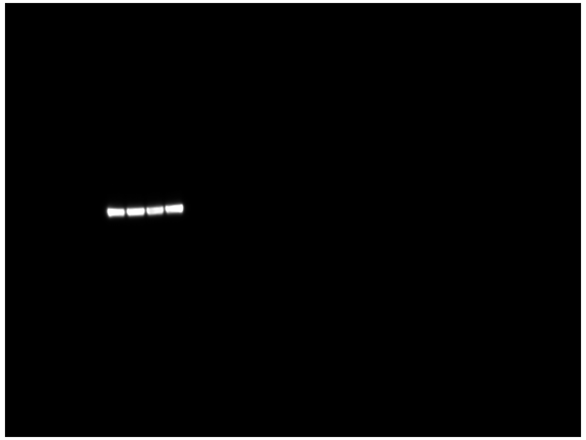
β-Tubulin
55KD

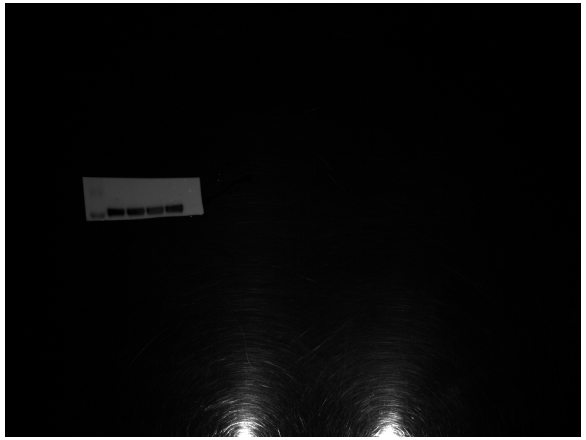
β-Tubulin with marker version
55KD

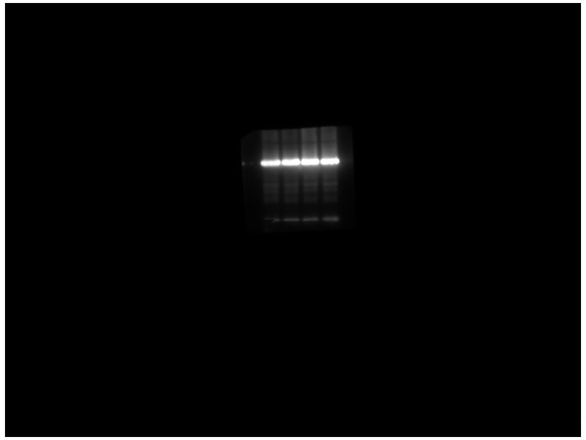
GAPDH
37KD

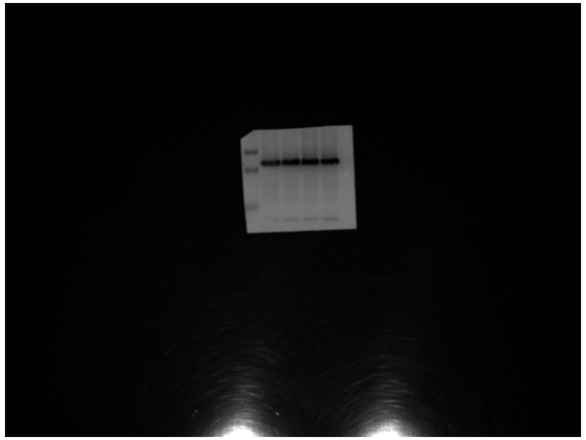
GAPDH with marker version
37KD

The original Gels of Fig. 5N and Fig.5O were presented.
